# Moderated digital social therapy for young people with emerging mental health problems: A user-centered mixed-method design and usability study

**DOI:** 10.3389/fdgth.2022.1020753

**Published:** 2023-01-09

**Authors:** Marilon van Doorn, Anne Monsanto, Cato M. Boeschoten, Thérèse van Amelsvoort, Arne Popma, Ferko G. Öry, Mario Alvarez-Jimenez, John Gleeson, Monique W. M. Jaspers, Dorien H. Nieman

**Affiliations:** ^1^ Amsterdam University Medical Centers (Location AMC), Amsterdam, Netherlands; ^2^ Department of Psychiatry and Neuropsychology, Maastricht University, Maastricht, Netherlands; ^3^ Buurtzorg Jong, Almelo, Netherlands; ^4^ Centre for Youth Mental Health, The University of Melbourne, Parkville, VIC, Australia; ^5^ Orygen, Parkville, VIC, Australia; ^6^ Healthy Brain and Mind Research Centre and School of Behavioural and Health Sciences, Australian Catholic University, Melbourne, Australia; ^7^ Department of Medical Informatics, Amsterdam Public Health Research Institute, Amsterdam UMC-Location AMC, University of Amsterdam, Amsterdam, Netherlands

**Keywords:** usability, user experience, indicative prevention, youth mental health, e-health, early detection, early intervention

## Abstract

**Introduction:**

Over 25% of Dutch young people are psychologically unhealthy. Individual and societal consequences that follow from having mental health complaints at this age are substantial. Young people need care which is often unavailable. ENgage YOung people earlY (ENYOY) is a moderated digital social therapy-platform that aims to help youngsters with emerging mental health complaints. Comprehensive research is being conducted into the effects and to optimize and implement the ENYOY-platform throughout the Netherlands. The aim of this study is to explore the usability and user experience of the ENYOY-platform.

**Methods:**

A user-centered mixed-method design was chosen. 26 young people aged 16–25 with emerging mental health complaints participated. Semi-structured interviews were conducted to explore usability, user-friendliness, impact, accessibility, inclusivity, and connection (Phase 1). Phase 2 assessed usability problems using the concurrent and retrospective Think Aloud-method. User experience and perceived helpfulness were assessed using a 10-point rating scale and semi-structured interviews (Phase 3). The Health Information Technology Usability Evaluation Scale (Health-ITUES; Phase 1) and System Usability Scale (SUS; Phase 2 and 3) were administered. Qualitative data was analyzed using thematic analysis. Task completion rate and time were tracked and usability problems were categorized using the Nielsen's rating scale (Phase 2).

**Results:**

Adequate to high usability was found (Phase 1 Health-ITUES 4.0(*0.34*); Phase 2 SUS 69,5(*13,70*); Phase 3 SUS 71,6(*5,63*)). Findings from Phase 1 (*N* = 10) indicated that users viewed ENYOY as a user-friendly, safe, accessible, and inclusive initiative which helped them reduce their mental health complaints and improve quality of life. Phase 2 (*N* = 10) uncovered 18 usability problems of which 5 of major severity (e.g. troubles accessing the platform). Findings from Phase 3 (*N* = 6) suggested that users perceived the coaching calls the most helpful [9(*0.71*)] followed by the therapy content [6.25(*1.41*)]. Users liked the social networking aspect but rated it least helpful [6(*2.1*)] due to inactivity.

**Conclusion:**

The ENYOY-platform has been found to have adequate to high usability and positive user experiences were reported. All findings will be transferred to the developmental team to improve the platform. Other evaluation methods and paring these with quantitative outcomes could provide additional insight in future research.

## 1. Introduction

Worldwide, one in seven young people (12–25 years old) experience mental ill-health (1–4), and the COVID-19 pandemic appears to have even further exacerbated the increase over the past few years in mental health complaints (5, 6). These complaints are the leading contributor to the burden of disease in young people (7, 8) and are associated with chronicity, lower social and occupational health, increased risk for suicide, self-stigma, decreases in quality of life and lost potential (8–12). Moreover, mental health complaints make up for the highest source of global economic burden (13, 14), namely an estimate of USD 2.5 trillion a year (15). In 2021, the Netherlands had the highest percentage of mental health complaints (depression, loneliness, anxiety and/ or stress) ever recorded. More than a quarter of Dutch young people were found to be “mentally unhealthy” [e.g., scored below 60 on the Mental Health Inventory 5, a screening instrument for complaints of depression, loneliness, anxiety and/or stress (1, 16)]. Despite the severity and impact of mental health complaints, clinical treatment is often not accessible in the Netherlands due to growing waiting lists for these clinical services (17–20) which, unfortunately, are projected to increase (19).

Fortunately, several initiatives are now being developed and implemented in and outside mental health care settings to intervene in an earlier stage (indicative prevention), namely when young people experience *emerging* mental health complaints. The goal of these initiatives is to prevent the worsening of mental health complaints and need for further mental health care. This strategy has already resulted in several creative and accessible mental health initiatives worldwide [e.g., (21–24)]. In Australia, since 2006, McGorry and his colleagues (25) have successfully implemented accessible Headspace centers for young people experiencing emerging mental health complaints (22, 25, 26). Research showed that Headspace significantly reduced psychological distress and improved quality of life and improved or maintained these positive changes at 2-year follow-up (22) as well. A recent review also found support for *online* indicated prevention interventions for youth Online interventions aim to improve access for underserved and difficult-to-reach populations like youths, as these interventions are reported to be more convenient than face-to-face interventions with regards to time, location and anonymity (27). Further, guidance has proven to be a beneficial feature of online mental health interventions (28). Amongst these online interventions, the *Moderated Online Social Therapy* (MOST) platform (29). Amongst them, the *Moderated Online Social Therapy* (MOST) platform (30), provides online care for youth with mental health complaints using evidence-based therapy exercises, private sessions with clinical moderators (psychologists) and peer workers (youth with lived experience with mental health complaints), and social support from the platform's community. Research findings from these randomized controlled trials showed that the implementation of MOST in Australia's national mental health care system resulted in increased vocational and educational recovery, reduced rates of hospital admissions and visits to emergency services, and had high levels of feasibility, acceptability, engagement and safety (31, 32). Several pilot studies across the diagnostic and severity spectrum and phases of treatment showed significant improvements in psychological distress, perceived stress, psychological well-being, depression, (social) anxiety, loneliness and suicidal ideation [e.g., (30, 33–35)].

The Netherlands has been the first country to implement the MOST platform outside of Australia, which commenced in 2018 under the name ENgage YOung people earlY [ENYOY; (36, 37)]. The Australian MOST platform was translated and adapted in cooperation with Dutch experts (researchers, clinicians, and peer workers) to fit the needs of Dutch young people. Through the secure and anonymous online platform young people could work on their mental health wherever and whenever they wanted, and were supervised and supported by psychologists and peer workers.

Comprehensive research is being conducted in order to assess the effects of the platform as well as to optimize and implement the ENYOY-platform throughout the Netherlands. Using quantitative methods, the effects on psychological issues, positive health and functioning are measured in a prospective cohort of 125 young people (16–25 years) with emerging mental health complaints in a mixed-method within-subjects study design. The overall goal is to optimize and implement the platform throughout the Netherlands (36, 37). The study is ongoing and the platform is under continuous development and co-creation (38) with involvement of the participating young people (36).

According to the World Health Organization (39), there is great potential in online mental health services when it comes to improving universal health coverage, provided these services are evidence-based (39). In order to refine and optimize an online mental health service such as ENYOY, the assessment of its usability is therefore recommended (40). A reason for conducting usability studies is that poor usability and lack of user-centered designs can result in low engagement and attrition (40). Additionally, usability studies help researchers to gain an understanding of how easy it is to use an online service, and to provide suggestions for change to safeguard this ease of use (40). Many electronic health interventions (eHealth) have already been subjected to usability assessment (41–43), applying methods such as semi structured interviews, questionnaires and focus groups. These studies yielded useful feedback and resulted in significant suggestions for improvement. Therefore, the current study is adopting a similar approach.

Qualitative research is also crucial for refining and optimizing complex designs (44). It contributes to in-depth feedback about platform design and functioning (45, 46); provides information about the user experience and satisfaction with the platform (47); could contribute to the ease of use of the platform; and could address usability problems (48).

Different qualitative research methods contribute to achieving these aims. A commonly used method is the semi-structured interview. Interviews add value by openly exploring user experience in more detail, as they encourage communication about individuals' needs and provide explanations for these needs. A pitfall of the semi-structured interview is that leading questions may result in socially desired responses (49). To overcome this disadvantage, another qualitative method that is commonly used is the Think Aloud (TA)-method. With TA, user feedback is obtained by letting participants “talk aloud” while performing or solving a task. TA aims to provide information about the cognitive thought processes of users while they interact with an application (47, 50). The TA method is most commonly used for specific usability problem detection, concerning the ease of navigation and intuitiveness of the digital intervention at hand. As it is task focused, social desirable responses are minimized (47). Combining these methods in a mixed method model could prove to be valuable in providing both standardized and unique data to provide an overall and detailed assessment of the usability and user experience of the ENYOY-platform.

The aim of this study is to gain a better understanding of the usability and user experience of the ENYOY-platform. The objectives are threefold: (1) to explore the usability, user-friendliness, impact, accessibility, inclusivity, and connection among peers as perceived by the end users, (2) to identify usability problems and give insights into possible causes, and (3) to investigate the user experience and perceived helpfulness of the ENYOY-platform.

## 2. Methods

### 2.1. Study context

This study took place within the context of the ENYOY-project [for a comprehensive overview, see (36)]. Young people (aged 16–25 years) with emerging mental health complaints used the ENYOY-platform for a total of 6 months. ENYOY was designed to support young people's autonomy in reducing their mental health complaints *via* personalized digital interactive psychological interventions in combination with professional online counseling with a clinical moderator (psychologist) and/or peer worker. Additionally, a moderated virtual support network is fully integrated and supports young people if and when they need it during their recovery journey (32, 35, 36, 51).

The ENYOY-project received ethical approval from the Medical Ethics Review Committee at Amsterdam University Medical Centers, the Netherlands (reference: NL66345.018.18), and was registered in the Netherlands Trial Register (ID NL8966). Written informed consent was obtained from all participants before inclusion to the study.

### 2.2. Study design

**Figure 1:**
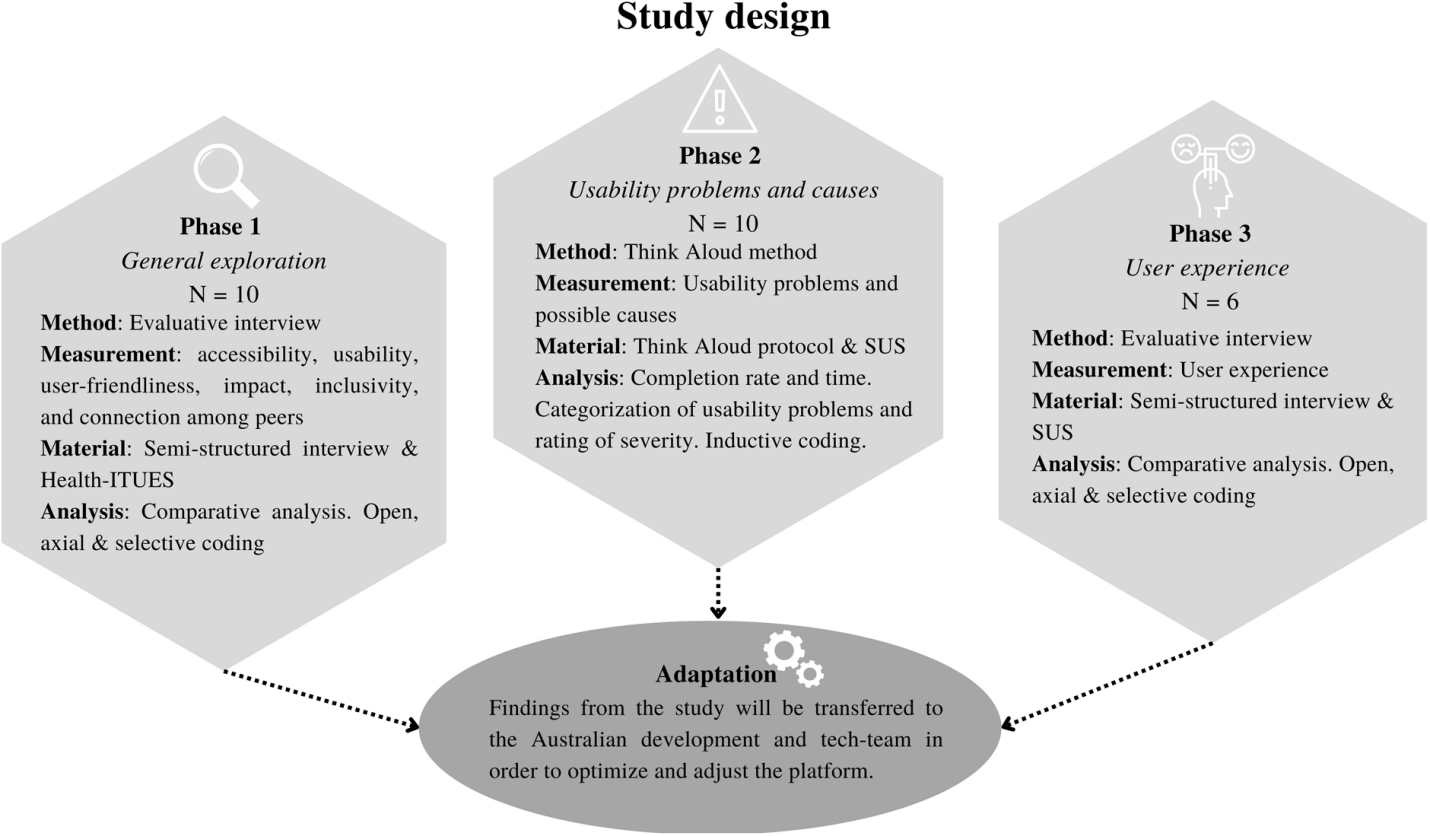
Study design. N, amount of participants; SUS, System Usability Scale; Health-ITUES, Health Information Technology Usability Evaluation Scale. For categorization of usability problems see Krushnik & Patel (2004), for Nielsen Severity Scale, see Nielsen (1994) in “Data analysis—phase 3”.

A user-centered (52) mixed-method design was used consisting of three phases, see Figure 1. In the first phase (*General exploration*), evaluative interviews were administered to explore the usability, user-friendliness, impact, accessibility, inclusivity, and experience of connection as perceived by the end users. In the second phase (*Usability problems and causes),* usability problems and possible causes were assessed using the concurrent and retrospective TA method. The concurrent TA method has been found to have higher effectiveness in terms of system redesign, whereas the retrospective TA method is the most optimal in finding user-customized design issues (47). In the third phase (*User experience),* the user experience and perceived helpfulness of the ENYOY-platform and its functions was assessed by evaluative interviews among participants. The Statement on Reporting of Evaluation Studies in health informatics framework was adopted by following the checklist of requirements per article section (e.g., title, abstract, keywords, introduction etc.) to ensure a good understanding of the study flow with the different phases (53).

### 2.3. Participants

A total of 26 young people (recently) active on the ENYOY-platform participated (see recruitment), of which 10 in phase 1, 10 in phase 2, and 6 in phase 3. For demographic characteristics, see Table 1. All participants were between 18 and 25 years old (M(*SD*) = 22,3(2,15)) and had emerging mental health complaints, as categorized by the clinical staging model stage 1a (mild symptoms and mild functional impairment) or 1b (attenuated syndromes with partial specificity with mixed/ambiguous symptoms and moderate functional impairment; see (54); and (36) for the operationalization in the ENYOY-study). 88,5% of the participants were female and 92,3% attended or graduated from higher vocational education or university. Participants had been involved in the ENYOY-project between 2 and 12 months (M(*SD*) = 8(*2,77*)) and on average spent 55,9 (*SD* = 42,6) minutes per week on the platform.

**Table 1:** Demographics per study phase and overall.

	Phase 1	Phase 2	Phase 3	Overall
Number of participants	10	10	6	26
Age, M (*SD*)	23.0 (*3.54*)	21.8 (*3.16*)	20.0 (*6.15*)	23.3 (*2.15*)
Clinical stage, *N* (%)				
1a	3 (30.0)	4 (40.0)	4 (66.7)	11 (42.0)
1b	7 (70.0)	6 (60.0)	2 (33.3)	15 (58.0)
Gender, *N* (%)				
Female	8 (80.0)	9 (90.0)	6 (100.0)	23 (88.5)
Male	2 (20.0)	1 (10.0)	0 (0.0)	3 (11.5)
Education, *N* (%)				
University	5 (50.0)	4 (40.0)	5 (50.0)	(46.2)
Higher voc. ed.	4 (40.0)	5 (50.0)	5 (50.0)	(46.2)
Intermediate voc. ed.	1 (10.0)	1 (10.0)	0 (0.0)	2 (7.7)
Ethnicity, *N* (%)				
Dutch	10 (100.0)	6 (60.0)	5 (83.3)	21 (80.8)
Surinam	0 (0.0)	3 (30.0)	0 (0.0)	3 (11.5)
Dutch + other	0 (0.0)	1 (10.0)	1 (16.7)	2 (7.7)
Platform usage (months)				
M(*SD*)	–	6.9 (*2.92*)	9.7 (*3.13*)	8 (*2.77*)
Range	–	2–10	8–12	2–12
Platform usage (m. *p*/w)				
M(*SD*)	–	52.5 (*38.89*)	61.7 (*51.45*)	55.9 (*42.6*)
Range	–	15–150	5–120	5–150
Used media, *N* (%)				
Laptop	–	3 (30.0)	4 (66.7)	7 (43.8)
Smartphone	–	3 (30.0)	1 (16.7)	4 (25.0)
Both	–	4 (40.0)	1 (16.7)	5 (31.2)

M, mean; SD, standard deviation; voc. ed., vocational education; m. *p*/w, =minutes per week; other, Tunisian/German/French.

2 = Messages of other young people, moderated by peer workers. 3 = The “Talk It Out” functionality. 4 = Hashtags for quick access to relevant subjects.

## 3. Materials

### 3.1. Treatment

**Figure 2:**
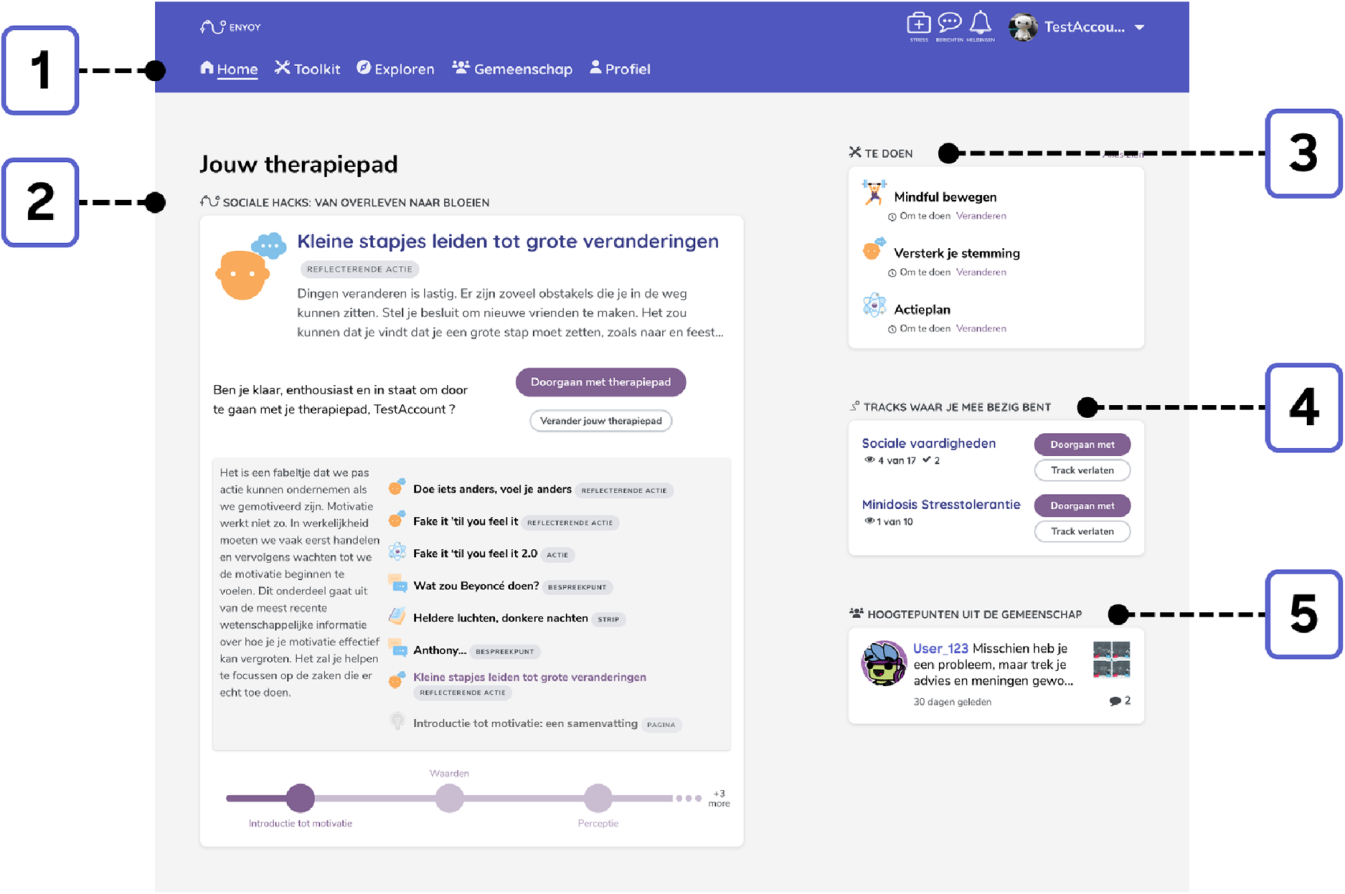
Exemplary homepage. 1 = Navigation through ENYOY (Home / Toolkit / Explore function / Community / Profile); 2 = Personalized therapy journey with a summary of next exercise;| 3 = To do list consisting of previously saved exercises (e.g. mindfulness); 4 = Overview of started stand-alone tracks regarding certain subjects (e.g. social skills); 5 = Highlighted messages in the Community.

Guided evidence-based (i.e., cognitive behavioral therapy, mindfulness-based cognitive therapy, social-cognition strategies) therapy journeys with a specific focus (anxiety, social anxiety, social function or depression) comprised a major component of ENYOY (Figure 2). An algorithm provided users with content based on their complaints and strengths. The activities within each journey consisted of: (1) therapy comics, which provide a playful insight into mental health complaints, (2) talking points, where one can share with the community what is on one's mind, (3) scientific pages, which introduced/summarized a topic relating to mental health (e.g., rumination), aiming to challenge stigma and provide psycho-education based on the latest scientific knowledge, (4) actions (regular, reflective, strength-based, and repeated) designed to help build mental health related skills (e.g., mindfulness) and reflect on challenges. Additionally, the explore function facilitated searches for exercises outside the current journey based on a struggle/need (e.g., stress or motivation) (30, 36).

Second, professional online support was provided. A clinical moderator was assigned to each participant to guide them through the platform *via* regular fortnightly coaching sessions through video calls. Additionally, all users were offered peer-to-peer support by a peer worker (30, 36).

**Figure 3:**
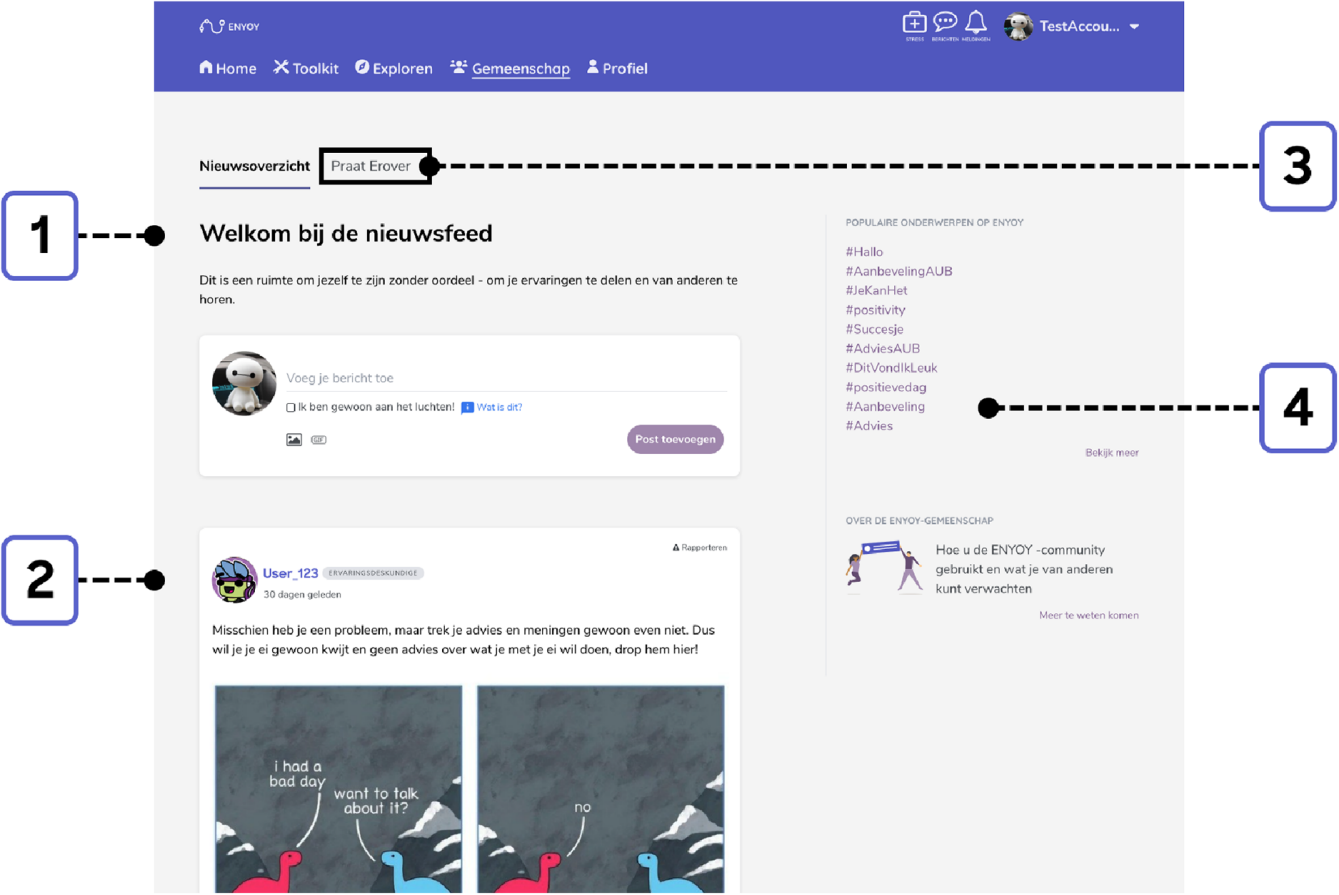
Exemplary community. 1 = Newsfeed and option to add a message to the Community.

The third element is the safe online social network, the “Community” (Figure 3). This is an online support network, moderated by peer workers, where individuals can share their experiences with other users. Users were encouraged to share messages and respond to messages from other young people in the community, but were not obliged to engage. Another part of the community is the Talk it Out-functionality (see Figure 3), where participants can ask peers for help and jointly brainstorm their problems. This function was not operational during the course of the study (30, 36).

**Figure 4:**
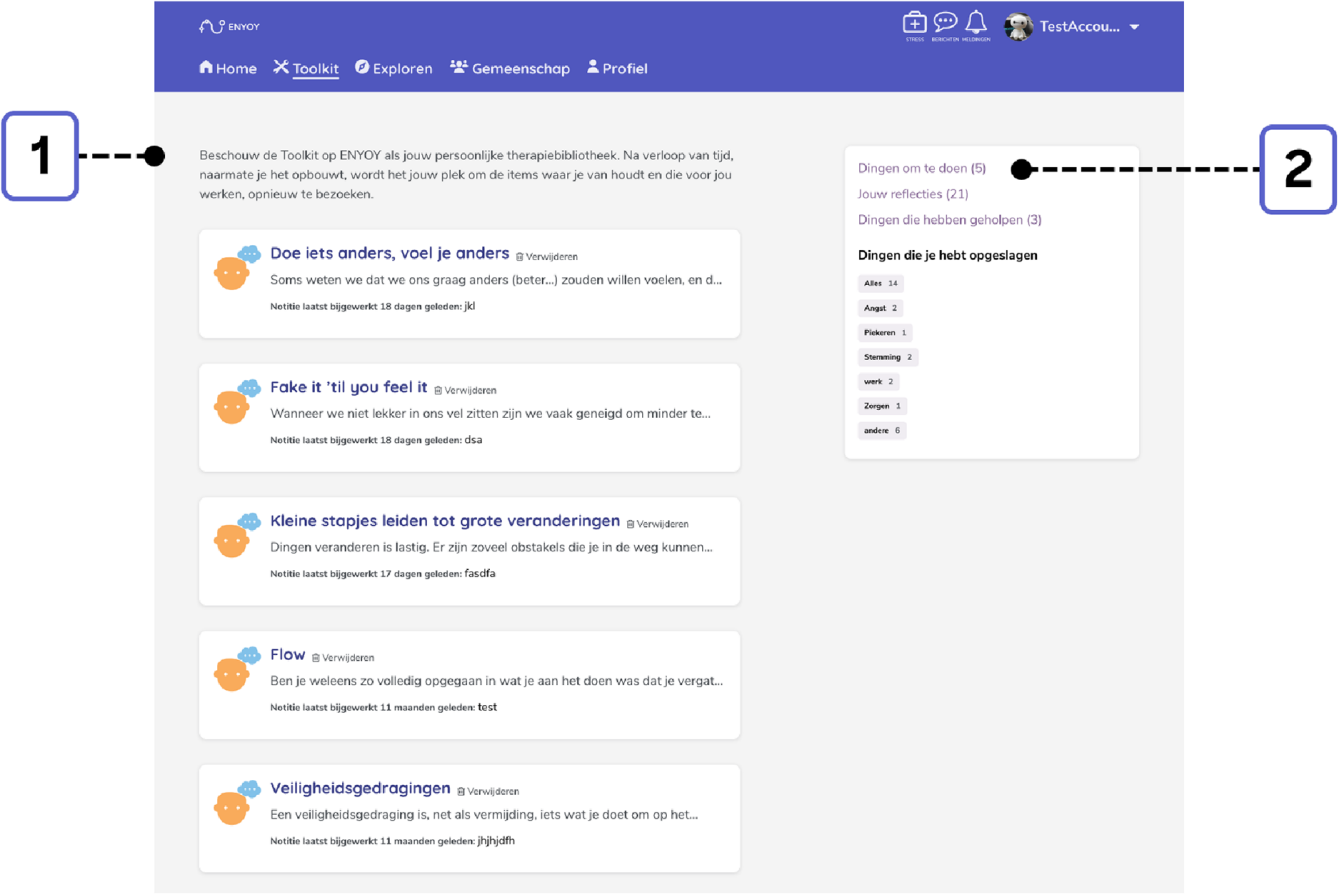
Exemplary toolkit. 1 = Overview of saved exercises. 2 = Saved exercises with personalized labels (e.g. anxiety, rumination, mood).

Fourth, a personalized therapy toolkit was available to save activities that were deemed helpful (see Figure 4). Young people could compile this personal library according to their preferences and could download any chosen content for later use (30, 36).

### 3.2. Interviews

All interviews took place one-on-one, *via* Microsoft Teams. The interviews in all phases took around 30 to 75 min. All interviews were voice recorded.

#### 3.2.1. Semi structured interview (phase 1)

The protocol for the semi-structured interview was constructed based on the relevant topics from the Health-ITUES e.g., usability, user-friendliness, impact, accessibility, inclusivity, and connection among peers (55), see Supplementary Appendix A1. An example question: “What was your experience with accessing the platform when you wanted to use it?” (accessibility). Open questions were formed and reviewed by experts in the field (MD, MJ).

#### 3.2.2. Semi structured interview (phase 3)

The protocol for the semi-structured interview was derived from the interview protocols that were performed for the MOST platforms in Australia (56), see Supplementary Appendix A2. The Australian protocol was translated in Dutch and questions that did not apply to ENYOY were removed or altered. Questions focused on the experience of the platform and its separate segments, e.g., the coaching calls, therapy exercises, the community, toolkit and explore function. For example, “What do you think about the community of ENYOY?” The interview protocol was reviewed by three experts in the fields of health technology, clinical psychology, social design and/or user experience research, one of them being an author of the current study (MD).

### 3.3. Usability evaluation method

#### 3.3.1. Think aloud-method (phase 2)

A TA-protocol was developed together with two experienced clinical moderators of the ENYOY-platform to ensure that the tasks in the protocol would be as realistic as possible and covered all functionalities of the “daily use” of the platform (47, 57); and consisted of a total of 8 tasks on the ENYOY-platform (see Supplementary Appendix A3). An example task was “Find tips on the platform on how to deal with stress”. This was the only phase in which the intervention was used by the participants as a frame of reference.

### 3.4. Questionnaires

#### 3.4.1. Health-ITUES

In phase 1, the impact, perceived usefulness, perceived ease of use and user control of the platform were assessed with the Health Information Technology Usability Evaluation Scale [Health-ITUES (55);]. The scale has yielded high internal consistency reliability (*a* = 0.85–0.92), moderate to strong criterion validity (*r* = 0.46–0.70) (58), and was translated back and forth (EN-DU) by four independent translators. The scale consists of 20 statements (e.g., “I think ENYOY is a positive addition for young people with emerging mental health complaints”) which were scored on a 5-point Likert scale (1 = strongly disagree, 5 = strongly agree). High scale values [>3.63; (59)] indicate a high perceived usability (58).

#### 3.4.2. SUS

In phase 2 and 3, the Dutch version of the System Usability Scale [SUS; (60)] was used; a 10-item questionnaire scored on a 5-point Likert scale (1 = strongly disagree to 5 = strongly agree), measuring the usability and acceptance of an intervention. The reliability of the scale is high (*a* > 0.90) and the concurrent validity acceptable (61). An example of an item is “I think that I would need the support of a technical person to be able to use this system”. The composite measure range is 0–100. Scores above 50 indicate acceptance (62).

### 3.5. Recruitment

All participants were recruited from the active users participants-pool of the ENYOY-project. The number of eligible users in the pool varied across the phases of this study. Phase 2 and 3 were conducted half a year later than phase 1; and less users were online on the platform at that time. Users could only participate in one of the three phases of this study. In Phase 1, users (*N* = 10) were randomly selected out of the participant pool (*N* = 106) and asked to participate. Whenever a participant was unavailable, the remaining eligible participants were again randomly assigned until the sample was complete. In Phase 2 and 3, all users were contacted *via* email or their clinical moderator because of the smaller participant pool (*N* = 40). Of these individuals, 16 had the time and interest to participate and were randomly assigned to either phase 2 (*N* = 10) or 3 (*N* = 6).

### 3.6. Procedure

An appointment was made through a secure Microsoft Teams environment. Interviews (30–60 min) were recorded with participants' permission using an offline recording device.

#### 3.6.1. Phase 1

During the semi-structured interview, participants were asked to fill out the Health-ITUES (55). Subsequently they were prompted by open-ended questions to share their views on usefulness, usability, accessibility, inclusivity, connection and contribution of the platform to the lessening of mental health complaints and increase of positive mental health. Participants were not asked to use the intervention during the interview.

#### 3.6.2. Phase 2

During the TA, participants were asked to perform 8 predetermined tasks on the ENYOY-platform while verbalizing their thoughts. The participant was randomly allocated to either the concurrent (CTA) or retrospective (CTA) TA condition. In the CTA condition, the participant was asked to describe every step when performing a task. In the RTA condition, the participant was asked to think aloud after performing the task (47). When participants did not understand the task, prompts were given by the research assistant. All audio was recorded and the participants' navigation patterns through the ENYOY-screens were video-recorded on an offline recording device. After the TA, the participant filled out the SUS (60).

#### 3.6.3. Phase 3

During the semi-structured interview, participants were asked to share their views on the value of the different segments of the ENYOY-platform (e.g., therapy journey, explore function, toolkit, community and coaching calls) by means of open-ended questions. They were not offered use of the intervention during the interview. Participants were asked to rate the platform and its segments on a 10-point scale (1 = “Not helpful at all” to 10 = “Very helpful”). After the interview, the participant was asked to fill out the SUS (60).

### 3.7. Data analysis

#### 3.7.1. Phase 1 and 3

All qualitative data from the semi-structured interviews was analyzed using deductive thematic analysis. Predetermined themes, as assessed in the interviews, were formed into categories. Data that did not fit these predetermined categories were further coded openly using inductive coding (63). Finally, selective coding was used to identify possible relations and connections between the data and categories (64). ATLAS.ti Windows (v22.0.6.0) was used to complete our work. Health-ITUES and SUS means and ranges were calculated.

#### 3.7.2. Phase 2

Task completion rate and time were tracked in order to obtain insight into efficiency. Usability problems were categorized into problem types (65): *usefulness*, whether the system provides meaningful, up-to-date, or valuable information to the user, and *ease of use*, which links potential difficulties or issues to the user interface or system design. Data that did not fit these predetermined categories was analyzed using inductive coding in order to form possible additional (sub)categories (63). All usability problems were rated using the Nielsen severity scale (66): 0 – I do not agree that this is a usability problem at all; (1) cosmetic problem only: need not to be fixed unless extra time is available on project; (2) minor usability problem: fixing this should be given low priority; (3) major usability problem: important to fix, so should be given high priority; (4) usability catastrophe: imperative to fix this before the product can be released). SUS means and ranges were calculated.

## 4. Results

### 4.1. Phase 1. General exploration

Results from the Health-ITUES yielded an overall high usability score [M(*SD*) = 4.0(0.34)], indicating that participants deemed the ENYOY-platform to be a highly usable technology (58, 59). Participants rated the impact the highest (*M* = 4,3) (i.e., the potential of ENYOY to strengthen the ability to cope with mental health complaints of individuals who use it), followed by the ease of use (*M* = 4,0) (i.e., absence of technical difficulties), user control (*M* *=* 3,9) (i.e., knowing how to deal with technical difficulties), and usefulness (*M* = 3,8) (i.e., the extent to which ENYOY has helped them with their mental health complaints).

#### 4.1.1. Semi-structured interviews

Several themes were subtracted from the interview protocol which were complemented with themes following from open coding of the data of the first two interviews. Axial coding showed overlap between several categories which were combined under one item. Finally, selective coding yielded the following six themes, (1) usability and user friendliness, (2) usefulness or impact, (3) inclusivity, (4) connection with others, (5) motivation, and (6) suggestions for improvement.

##### 4.1.1.1. Usability and user friendliness

Most participants [6]^1^ found the platform easy to use and described a good level of usability. Participants thought the platform was “user friendly” [2], that it provided a clear overview [2], looked neat [1], offered a distraction from personal struggles [1], had a great lay-out [1], and that everything worked well [1]. One participant mentioned they^2^ hardly ran into problems, and if they did, their clinical moderator helped them out quickly.

*[P1]: “I think it [red: ENYOY] is quite comprehensible. It is very user friendly. I really think the therapy journey you are assigned to has a logical order. […] The combination of separate exercises and a specific journey with customized exercises really helps. […] It is easy to find everything.”* ^*3*^

Other comments that were made were “easy to use” [6], “simple” [6] “comprehensible” [2], “accessible” [5] and “lots of (exercise) choice” [2]. Two participants mentioned the platform being a bit repetitive, because they already were acquainted with some of the theory and exercises on ENYOY because of receiving previous psychological therapy. Five participants described having difficulties using the two-step authentication, which was necessary for privacy protection (36). Three participants mentioned they felt a bit annoyed when content was not fully translated from English to Dutch yet or when they came across spelling errors, which made one participant think that the website was automatically translated. Other challenges were “the inability to go back to previous exercises” [2], “having to request a switch to another therapy journey” [3] and “being automatically assigned to a journey by the algorithm of the platform” [2].

##### 4.1.1.2. Usefulness or impact

Six participants mentioned that ENYOY helped them reach their therapy goals and seven noticed a clear improvement of their positive health.

*[P5]: “I do still experience stress, for example, but I find it easier to sit down and make it go away in one minute instead of ten minutes.”*

Others [6] reported that ENYOY felt like a “social safety net” or a system that made them feel supported and heard. Three participants mentioned that due to the platform, they were allowing themselves to pay attention to and become more aware of their mental health complaints.

*[P6]: “I like it because, eh yeah … It makes you feel like you’re moving in the right direction and that you are paying attention to it. With something that’s really made for it. And not like, yeah* *…* *all these separate, slightly vague articles that you find on the internet.”*

Three participants mentioned that the platform did not so much contribute to the improvement of their positive health, either because they felt as though they did not need much improvement in that area [2], or because they did not put enough effort into the platform [1]. Three participants mentioned they found it difficult to incorporate the platform's tips and tricks into their daily lives.

##### 4.1.1.3. Inclusivity

None of the participants reported any problems relating to inclusivity. Five participants mentioned they felt accepted, welcome, included and/or taken seriously by the other young people on the platform. Seven participants noticed that there always was someone replying to their or other people's messages in the community. Other comments were that people were “respectful” [1] and that it was “nice to be around people with similar problems” [2]. One participant mentioned they especially felt welcome by reading other people's messages on the community feed.

*[P7]: “People were very open, despite it being quite sensitive topics […] I liked it, it felt very inclusive to read.”*

##### 4.1.1.4. Connection with others

All interviewees reported a positive clinical moderator experience. They felt able to share personal information and were happy with the suggestions they received. Two participants had spoken to a peer worker, which resulted in a positive experience, and one participant had talked to another person on the platform in a similar domestic situation. Four participants were explicitly positive about sharing messages in the community and the others indicated they preferred reading the messages from others instead of sharing themselves.

*[P5]: “I find it hard to talk about my problems* *… so ENYOY was already a big step for me […]. That's why I didn't really want to join the community and just wanted to keep it as low-key as possible for myself, because I prefer it that way.”*

Although participants understood that chatting with other participants was not possible due to privacy measures, they would have preferred to chat with their fellow ENYOY-members [4].

##### 4.1.1.5. Motivation

Participants mentioned various reasons for joining ENYOY. Half did not receive enough help from their (previous) psychologist/GP and four indicated they were hesitant to see a psychologist. Other reasons were that ENYOY “has no waiting lists” [4], “is easy to do from home” [2], “has a low threshold” [2], “is free” [1] and “respects your autonomy” [2].

*[P10]: “I really needed help back then, but that seemed like quite a big step, hence this [red: ENYOY] appeared to be a great solution […] and with all the long waiting lists in the Netherlands and eh, in mental healthcare, it's just disastrous* *… And it's a study of course, and the initiative seemed great and nice and very accessible for young people.”*

#### 4.1.2. Suggestions for improvement

Several suggestions were given (Supplementary Appendix A4), e.g., adding notifications to help motivate users to spend more time on ENYOY, or enabling direct contact with other users *via* the platform.

### 4.2. Phase 2. Usability problems and causes

The perceived usability of the ENYOY-platform as measured by the SUS was 69,5 (SD = 13,70). This score indicates that the ENYOY-platform is considered to have adequate usability.

#### 4.2.1. Task completion

For participants' completion rates and times, see Supplementary Appendix A5. All tasks were fully completed in the CTA, except for one participant who did not complete task 5. Participants in the RTA group only fully completed tasks 1 and 7. On average, task 1 and 2 took longest to complete in both groups (T1: CTA: M = 344s(*236*) & RTA: 148s(*106*); T2: CTA: 106s(*51*) & RTA: 147s(*56*)) which might indicate the presence of usability problems in the steps within these tasks. The task with the shortest completion time and full completion rate was task 7 (CTA: M = 21s (SD = 12) & RTA: 29s (31)).

#### 4.2.2. Usability problems and bugs

A total of 18 usability problems were found (see Table 2; for a full overview see Supplementary Appendix A6). Five usability problems were rated with a severity of “3” (major usability problem), 12 with “2” (minor usability problem), and one with “1” (cosmetic usability problem). None were found to be of catastrophic severity (4), indicating that no problem is required to be resolved with high urgency (66). The CTA group found seven unique problems and the RTA group three. During the TA method two bugs were found: (1) an internal server error in the toolkit and (2) the platform froze for a short time.

**Table 2:** Overview of distinct usability problems detected per method and rating of severity.

Severity	CTA	RTA	CTA and RTA	Total
1	1	–	–	1
2	5	3	4	12
3	1	–	4	5
4	–	–	–	0
Overall	7	3	8	18

For the distribution of usability problems by problem type and severity, see Table 3. The CTA showed higher sensitivity in detecting unique terminology (*N* = 2) and navigation (*N* = 3) problems, and the RTA revealed more overall ease of use (*N* = 1) and unclear graphics or symbols (*N* = 1) problems. Usability problems of almost every problem type were found. Overall, the CTA detected more usability problems (*N* = 15) than the RTA (*N* = 11). Most were navigation problems (CTA = 6, RTA = 3), indicating that participants had difficulties finding desired information on the platform. The most severe problems (severity = 3) considered visibility, overall ease of use, terminology interpretation, and navigation problems. The next section will provide more insight into the most severe usability problem(s) (severity 2 & 3) per aforementioned type.

**Table 3:** Distribution of usability problems by problem type and severity per TA method.

Problem type	Total		CTA/RTA (*n* = 8) Severity				CTA unique (*n* = 7) Severity				RTA unique (*n* = 3) Severity			
	CTA	RTA	1	2	3	4	1	2	3	4	1	2	3	4
Visibility problems	3	2			1		1		1			1		
Overall ease of use	1	2			1							1		
Errors/ help instructions	1	1		1										
Terminology interpretation problems/meaning of labels	4	2		1	1			2						
Unclear graphics/symbols	–	1										1		
Navigation	6	3		2	1			3						
Total	15	11		4	4		1	5	1			3		

#### 4.2.3. Usability problems with the highest severity rate

##### 4.2.3.1. Visibility problems (severity = 3)

A problem was encountered in the system's two-factor authentication. Four participants filled their own personal login information in the data entry field instead of the general login information. This login problem could lead to no access to the platform.

##### 4.2.3.2. Overall ease of use (severity = 3)

During the first task several participants [7] could not find the ENYOY-platform without help from the researcher or email with the website link.

*[P6]: “I always opened it [red: ENYOY] via my mail and logging in took me half an hour*.”

Another severe ease of use problem was that some of the exercises on the platform were not (fully) translated into Dutch. This could cause comprehension issues [1].

##### 4.2.3.3. Terminology interpretation problems (severity = 2)

For the second task, participants were asked to request a different therapy journey without contacting somebody from the platform. Three participants found the choices (“adjust” or “change therapy journey”) confusing.

##### 4.2.3.4. Navigation (severity = 3)

In task 5, participants were asked to send a chat request to a moderator or peer worker. Six participants navigated to the community page instead of to “messages” because they expected to find this function there.

*(P9): “The first place I would look for a peer or an experienced expert is within the community.”*

#### 4.2.4. Suggestions for improvement

Several suggestions for improvement were given, see Supplementary Appendix A4. For example reducing text in exercises about stress, or adding a “search function” in the explore function.

### 4.3. Phase 3. User experience

The SUS measured a perceived usability of 71,6 (SD = 5,63) suggesting adequate usability. Participants rated the overall perceived helpfulness of ENYOY with a 7.67 (*SD* = 0.41), see Table 4.

**Table 4:** Average perceived helpfulness scores of the components of ENYOY on a 1–10 scale.

	Coaching calls	Journeys	Explore function	Toolkit	Community	ENYOY (overall)
Mean (*SD*)	9 (0.71)	6.25 (1.41)	5.67 (2.94)	5.08 (2.11)	6 (2.1)	7.67 (0.41)

Thematic analysis of the data yielded the following categories: (1) overall perceived helpfulness, (2) coaching calls, (3) exercises, (4) therapy journeys, (5) explore function, (6) toolkit, (7) community, and (8) suggestions for improvement.

#### 4.3.1. Overall perceived helpfulness

All participants were positive about ENYOY and considered it helpful. They indicated that they liked the range of options to work on their problems [2] and being anonymous [2]. Sometimes participants forgot ENYOY was available [4] and would only be reminded to do exercises right before a coaching call [2]. Two participants reported an occasional bug or translation error. Some requested a smartphone app [2] as logging on was difficult [3].

[P2]: *“I think I got to know myself better”*.

#### 4.3.2. Coaching calls

The helpfulness of the coaching calls was rated high [9 (*SD* = 0.71), Table 4]. Participants received direct feedback [2], found new insights into their issues [2], and suggested exercises were helpful [3]. Participants reported having a good connection with their clinical moderator [2] and were able to talk about their problems and feelings [2].

#### 4.3.3. Therapy journeys

The therapy journeys were rated above average, [6.25 (*SD* = 1.41), Table 4]. Most participants found the journeys helpful in relieving their symptoms [5]. The use of the journeys varied. Minimal or no use was mainly due to the journey not completely matching their needs [6], which was caused by the journey being too rigid, as you must follow specific exercises in a particular order [2], a mismatch with their current mental health complaints [1] or because having prior knowledge about their mental health complaints and how to solve them made it redundant [1].

[P2]: “*It [red: therapy journeys] gives a good start in helping yourself and makes it possible to evaluate things like: ‘How am I doing? What am I struggling with?’. Because of this, you discover things about yourself you did not know before.”*

#### 4.3.4. Explore function

Participants' opinions about the explore function were mixed [5.67 (*SD* = 2.94), Table 4]. Some found the function helpful and reported using it as their primary exercise source when the journeys did not fulfill their needs [2] Others indicated that they did not need it [3].

#### 4.3.5. Exercises

The exercises (found within the journey or *via* the explore function) were well-liked and found helpful [5]. Most participants reported not doing exercises often due to forgetting about ENYOY [4]. One participant reported the exercises being too long or consisting of too much text.

[P6]: “*…* *a couple of exercises really made a shift in my way of thinking, like, oh, I could also look at this in this way. And that helped me”*

[P5]: “*…* *If I wrote something down it was as if I had sent the thoughts away* *…* *and then it would be kind of gone. That is what I liked about the platform”*

#### 4.3.6. Toolkit

The toolkit was rated low, [5.08 (*SD* = 2.11), Table 4]. Participants indicated they did not use it often [4], but all liked the idea of saving helpful exercises. One reported not using it because of being preoccupied with finishing the journey and two reported using other means to save helpful exercises, e.g., writing them down. Some reported that they wished they had looked back at the end of the ENYOY-period at the saved exercises to self-reflect [2].

#### 4.3.7. Community

Most participants liked the community [5] and regarded it as a safe space [2]. Participants who shared a post [3] found the replies of the peer workers helpful [3]. Most reported the activity was too low [4]: they would have shared more if there were more active users [4]. The low rating [6 (*SD* = 2.1), Table 4], was because of the lack of activity and not because they did not find this function helpful.

[P1]: *“I really liked that you could just put things on there and that people gave sincere responses.”*

#### 4.3.8. Suggestions for improvement

Several suggestions for improvements were given by the participants. For example making a smartphone app for the platform, or make it possible to “go back” in the therapy journey to review completed exercises. For a full overview of suggestions, see Supplementary Appendix A4.

## 5. Discussion

The aim of this study was to gain a better understanding of the usability and user experience of the moderated online social therapy-platform ENYOY, following a user-center (45) mixed-method design framework.

In all phases, adequate to high usability of the ENYOY-platform was found. The findings from phase 1 (*General exploration*) indicated that users were very positive about ENYOY as a user friendly, safe, accessible, and inclusive initiative which helped them to reduce their mental health complaints and improve quality of life. Users indicated ENYOY helped them reach their therapy goals, improved their positive health, and felt like a social safety net. Users had positive moderator experiences and felt accepted, taken seriously, and welcome on the platform. Examples of pitfalls were that users missed the option to chat privately with other young people and some found it difficult to incorporate the platform into their daily lives. In phase 2 (*Usability problems and causes)* a total of 18 usability problems were found. Most of the problems had a minor severity level, meaning low priority should be given to resolve the problem. Five problems were found to be of major severity level and have a higher urgency to be resolved (e.g., troubles accessing the platform or a navigation problem in finding a way to chat with a peer worker). The findings of phase 3 (Phase 3 – *User experience)* showed that the users perceived the bi-weekly coaching calls with their clinical moderators as the most helpful to relieve their symptoms, since it helped them to see new perspectives and they received immediate feedback on their problems. Users rated the therapy content the second most helpful. While the exercises were found useful, not all of the exercises matched the participants' needs. The users indicated they liked the social networking aspect but rated it the least helpful due to the platform not being active enough for them to post themselves. The most relevant findings per phase and suggestions across phases will be further discussed and explained below.

In phase 1 some users indicated they would have preferred to have a say in the therapy journey they were assigned to. This is especially relevant when a therapy journey does not completely match the users' needs, which was the case for about one third of the users in phase 1 and all users in phase 3. Being “stuck” with a therapy journey could induce a decreased sense of autonomy when using the platform. MOST and ENYOY are largely based on the Self-Determination Theory (STD(67):) which states that when an individual senses that their need for self-government and self-control is not being met, their sense of well-being and competence diminishes. Conversely, increased autonomy could lead to greater feelings of self-efficacy and personal mastery (68) which has been shown to moderate motivation for improving one's own psychological health (67). It is therefore highly recommended that users are given more self-direction. For example, users could be allowed to switch between journeys without needing the permission of their clinical moderator. In phase 2, differences in individual usability ratings were large. Allowing for greater self-direction by including an option to tailor the platform to one's own needs, could also contribute to more stable usability ratings.

The results from phase 2 revealed that two tasks were the least efficient to perform: (1) logging on to the platform and (2) requesting a different therapy journey. This could indicate the presence of usability issues within these tasks, which indeed is in line with the TA results, and has priority to be resolved. Another interesting finding, in agreement with Kuusela and Paul (69), is that more unique usability problems were found in the CTA than the RTA-group. This could be explained by the limited capacity of short-term memory: If thoughts are shared *after* the experiment, some information will be lost, simply because one has forgotten part of the thoughts that occurred *during* the experiment (69). The difference in unique usability problems found by CTA vs. RTA indicate that the detection scope of these methods could vary. In accordance with (47), the CTA detected navigation problems that could be classified as system redesign problems and the RTA detected overall ease of use problems, which could indicate a user-customized design problem. The value of both methods for providing unique information about ENYOY is therefore underlined.

The findings of phase 3 showed large overlap with the findings of the Australian user experience study of MOST (56). Both studies found that users considered the therapy exercises to be helpful but consisting of too much text. The amount of text that is presented per exercise/webpage is highly relevant for digital therapy initiatives; the bigger the amount of text on webpages, the fewer will be read (70, 71). Decreasing text sizes could improve exercise completion rates and increase perceived helpfulness, and therefore possibly the effects of an intervention (72). Another way to improve this could be through gamification of the exercises or learning by “doing”. In this way gaming elements are added to a learning environment to further improve learning, for example by adding challenges and rewards such as badges or points (73).

Interestingly, the Australian young adults found the social networking aspect of the platform to be the most helpful while the Dutch found it the least. This could reflect a cultural difference. Another explanation is that during the Australian study the number of online users was higher. Dutch users reported feeling a threshold to post on the community because it was too inactive. The participation inequality principle of social media platform use states that 90% of users do not post, 9% respond to content, and 1% create new content (74, 75). If the platform has more active users, more content will be created and there will be more posts to respond to. Indeed, based on observations of the ENYOY-clinicians, community activity (a rough estimate of the number of posts per week) appears to increase at the threshold of 40 users. This could also explain why users in phase 1 seem more positive about the community, since at that time, the amount of users was above 40. Unfortunately, overcoming participation inequality is not possible. However, it could be improved, for example by letting users build from existing templates and rewarding them for their posts (75), as is the case for the “Talk It Out” functionality of MOST+. A “Talk It Out” lets a user define a problem and asks other users to brainstorm a solution to that problem. This function was rated the most helpful in Australia (56), but at the moment of conducting this study, was not yet implemented in the ENYOY-platform.

Several suggestions were mentioned by users across phases, emphasizing their relevance (Supplementary Appendix A4, Table a): a need for (1) insight into previously completed exercises; (2) uniformity of the platform in terms of language (e.g., Dutch instead of English); (3) addition of notifications to incorporate ENYOY into daily routine; and (4) simplify the log-in method (e.g., with easy-to-use two-step verification). According to the SDT (67), the first suggestion may be related to a decreased sense of competence: As users advance in the platform without being able to look back on their previously completed exercises and therapy journeys, they may forget what they have learned and their feelings of accomplishment diminish, which may lead to lower self-efficacy. MOST in Australia has a functionality which lets users “level up their talents” (e.g., curiosity, friendliness) based on the therapy exercises they perform and quantifying progress through the platform. This might provide a valuable solution for ENYOY. The second suggestion may be related to a decreased sense of competence and relatedness. Due to language barriers (e.g., not being able to understand text that has not yet been translated from English to Dutch), users might experience difficulty relating their own mental health issues to the exercise, which in turn may lower perceived usefulness and hinders their sense of competence and relatedness. The third suggestion may relate to a diminished sense of autonomy: as users tend to forget to use the platform in between coaching calls, notifications may serve as reminders for using the platform, increasing user autonomy. For example, a user could be given the option to set notifications (*via* email or text message) on/off if a user has been inactive for a certain amount of days. Lastly, the current log-in method may create friction in terms of user-friendliness, which decreases platform interaction and diminishes feelings of competency. Optimizing the log-in function to work friction-free may therefore be top priority, e.g., by two-way authentication by fingerprint, face-id or personal code.

Additionally, the following phase unique suggestions were given (see Supplementary Appendix A4, Table b). In phase 1, unprompted suggestions were mostly related to user friendliness of the Journey navigation, while in phase 2 prompted suggestions also arose about the ease of use of the explore function, the stress-button and community. This may signal that the therapy journeys are more widely used and more top-of-mind when users are asked about the platform, while other, lesser used functionalities (e.g., explore function or toolkit) might also require improvement. This is in agreement with the findings in phase 3; more than half of the users did not or seldom use the explore function or toolkit. Nevertheless, caution is needed when comparing and concluding about the suggestions between phases, since all phases used different questionnaires and methods. Interestingly, users across phases reported positive experiences with—and ease of use of the ENYOY-platform, even though several severe usability problems were found in phase 2. One possible explanation for this finding is that the amount of found usability problems may be in accordance with the “adequate to high” usability found within the quantitative data, and therefore possibly puts the found problems in a broader perspective of an overall good functioning platform. Another explanation for the seemingly large gap between usability problems found and rather positive perceived ease of use of ENYOY could be the coaching calls with clinical and peer moderators, which have not been taken into account in phase 2. This form of personal contact may have positively influenced the overall experience of the platform. In point of fact, previous research (29) on indicative prevention found that a combination of clinical or/and peer moderation with online therapy in digital interventions for young people resulted in the most stable and highest effect sizes.

### 5.1. Strengths and limitations

Several strengths and limitations of this study should be highlighted. One limitation relates to the representability of the samples in all phases. Given that interviews were voluntary, the more enthusiastic and motivated individuals might have signed up, hence resulting in bias when reporting their platform experience (76). Nonetheless, the significant amount of retrieved usability problems and suggestions offer a great starting point for improvement. Additionally, the gender was not equally distributed, which could affect the validity of the study. The higher number of participating females is similar to other digital indicative preventive mental health initiatives (29). Possibly, men tend to acknowledge mental health problems at a later or more advanced stage than women (77, 78), tend to experience more mental health stigma (79), or the content these initiatives offer might better match the complaints women experience (e.g., more internalizing vs. externalizing problems (80). Third, the difference in participants' platform experience based on time spent on the platform may have yielded a recall bias (81). Interviewees had a maximum of six months of not being on the ENYOY-platform, hence for those who have not been using it for a significant period of time (about one third of the participants were offline for four or more months), their autobiographical recollection and opinions may have changed as time passed and poses a risk of forgetting. Conversely, relatively new users (two users were online between two and three months) may not know all the functionalities of the platform, which also could have resulted in a lesser understanding. Another limitation was that the results of the study are time, place and platform specific: Our recommendations are to be taken with caution, as they have not been validated across MOST-projects, teams, or environments.

A strength of this study is its mixed methodology. According to the principle of method triangulation, the use of several methods could aid researchers in grasping the various layers of experience data and improve the multi-dimensionality of the study (82). As multiple qualitative methods were used in this study, the combination of standardized and unique data likely provides an overall and detailed assessment of the usability and user experience of the ENYOY-platform. Moreover, this qualitative study is deemed to enrich the quantitative research that is currently carried out, in relation to the ENYOY-platform (36, 37). Another strength was the sample size. Although phase 3 yielded less participants, it is expected that the overall study provided insight into at least 80% of the existing usability problems in the platform (50, 66). Additionally, with the aim of implementing ENYOY (or MOST+) worldwide, a study concerning the platform's intermediate usability and user experience could lay a strong foundation for future platform implementations, despite cultural differences. Finally, the ENYOY-platform has great potential to contribute to the prevention of more severe mental health disorders, and since it is an online platform, especially during the COVID-19 pandemic.

### 5.2. Future research

All usability and user experience issues will be addressed by the developmental team. We recommend continuing the investigation of the usability and user experiences after processing these. Additionally, research into gender differences in help-seeking behavior in individuals with emerging mental health complaints and how to better match indicative prevention initiatives with the needs of young men could lead to improvement of mental health of this group. Moreover, use of other evaluation methods in future studies of ENYOY could provide additional insights. For example, heuristic evaluation or cognitive walkthrough could uncover potential usability problems derived from general knowledge about how humans cogitate through tasks (83), and could be used in comparable versions of the ENYOY-platform. Research into other aspects of user experience could provide a broader scope and help create a platform that better fits the user's needs, e.g., user analytics (84) might offer quantitative insight into the user experience by what type of content is used the most, and focus groups could offer more insights into the users' desires (85). Lastly, it could be interesting to compare quantitative outcome measures with different usability or user experience aspects, e.g., is there a difference in the effect of the platform on mental health complaints for individuals who rate the usability high vs. low, or who experience the platform differently? And if so, how could this be explained and controlled for?

### 5.3. Recommendations

Overall, the ENYOY-platform has been found to have adequate to high usability and users reported positive experiences using it. All findings and recommendations of this study have been transferred to the development team in order to adjust and improve the platform. It is recommended to continually engage users during this optimization process. One serious problem considering the usability that should be underlined is the access to the platform. If young people have trouble finding and accessing the platform, their engagement may be jeopardized. This should be investigated for other versions of the platform and adjusted forthwith. Additionally, the platform might benefit specifically from providing users with more options to choose from themselves in order to enhance self-determination (68), e.g., decide on selected therapy journey, set notifications on/off, option to navigate “back” in therapy journey. ENYOY could be further optimized by adding the “Talk it out”, and “Level up strengths” functionalities. Finally, adding elements of gamification could improve learning and the use of the intervention (73).

Given that these recommendations are very platform-specific, it is challenging to draw any conclusions about the study's relevancy to other usability studies. Nonetheless, the aforementioned recommendations of ensuring proper engagement, platform access, sense of self-determination, autonomy and gamification can be taken into account when trying to improve other eHealth services through usability research.

## Data Availability

The raw data supporting the conclusions of this article will be made available by the authors, without undue reservation.

## Footnotes

^1^The amount of participants who provided the data on which a result is based, is showed between brackets.

^2^In order to preserve anonymity, participants’ names and gender are not reported.

^3^As interviews were conducted in Dutch, quotes were translated, reviewed by multiple researchers, and reported in English for the purpose of this article.
