# Supplementary material for: Moderated digital social therapy for young people with emerging mental health problems: A user-centered mixed-method design and usability study
Source: Front Digit Health. 2023 Jan 9;4:1020753. doi: 10.3389/fdgth.2022.1020753 (PMC9869113; doi:10.3389/fdgth.2022.1020753)
Supplement: Supplementary file 1 [file Datasheet1.docx]

**Appendix 1.** Semi-structured interview phase 1

All questions were translated from Dutch to English for the purpose of publication of this article.

Topic 1: usefulness

• ‘To what extent did you feel like the platform could help you reach your therapy goals?’

Topic 2: user friendliness/usability

• ‘What is your opinion on the user friendliness of the platform?’

• ‘Did you experience any problems using the platform?’

Topic 3: accessibility

•  ‘What was your experience with accessing the platform when you wanted to use it?’

• ‘Were you able to access all aspects of the platform?’

Topic 4: inclusivity

• ‘Can you tell me something about whether you felt accepted on the platform?’

• ‘Did you experience any issues in terms of acceptance on the platform?’

Topic 5: connection among peers

• ‘How did you experience the possibility of connecting with peers on the platform?’

• ‘In what way (if any) did you connect with peers on the platform?’

Topic 6: contribution of the platform to the lessening of mental health complaints and increase of positive mental health

• ‘In what way do you think the platform contributed to the lessening of your mental health complaints?’

• ‘In what way did the platform contribute to your positive mental health?’ (give examples: ‘daily functioning’, ‘quality of life’, etc.)

Topic 7: motivation

• ‘What was the reason you chose to sign up for ENYOY (i.e. compared to other mental healthcare services)?’ (this question also aims at the gender divide on ENYOY: only a few men have signed up for participation compared to a lot of women – we’d like to know why)

Topic 8: clinical moderators

• ‘How did you experience the coaching sessions with your clinical moderator’?

• ‘Do you feel like ENYOY can offer you enough when it comes to lessening your mental health complaints? If not, what is missing?’
