# Supplementary material for: Moderated digital social therapy for young people with emerging mental health problems: A user-centered mixed-method design and usability study
Source: Front Digit Health. 2023 Jan 9;4:1020753. doi: 10.3389/fdgth.2022.1020753 (PMC9869113; doi:10.3389/fdgth.2022.1020753)
Supplement: Supplementary file 2 [file Datasheet2.docx]

**Appendix 2.** TA-protocol Usability study

All text and tasks were translated from Dutch to English for the purpose of publication of this article.

| Task | | Description | Achieved when |
| --- | --- | --- | --- |
| 1 |  | Log in on ENYOY-platform  and hide profile and messages | User found the button to hide profile and messages and switched the button |
| 2 |  | Request a different therapy journey. | User found the questionnaire to change the therapy journey |
| 3 |  | Exercise search based on category and type. Find comics for ‘Piekeren’ via the explore function | Comic ‘Angstige gedachten voor de rechtbank’ or ‘De valse waarzegger’ has been found |
| 4 |  | Save exercise and find saved exercise | User successfully found the saved exercise in Toolkit |
| 5 |  | Find a way to chat with a random peer, moderator, or experience expert by sending a chat request | User found the ‘berichten’-button and the place to leave a message for a chat-request |
| 6 |  | Find suggestions on the platform when feeling distressed | User found the ‘stress’-button |
| 7 |  | Find a way to send a message in the Community without worrying, because of venting. | User found the 'Ik ben gewoon aan het luchten!'-button and selected it before posting a message on the Community. |
| 8 |  | Find a mindfulness exercise in the explore function. | User found the ‘3 mindful ademen’-exercise |
