# Supplementary material for: Moderated digital social therapy for young people with emerging mental health problems: A user-centered mixed-method design and usability study
Source: Front Digit Health. 2023 Jan 9;4:1020753. doi: 10.3389/fdgth.2022.1020753 (PMC9869113; doi:10.3389/fdgth.2022.1020753)
Supplement: Supplementary file 3 [file Datasheet3.docx]

**Appendix 3.** Semi-structured interview phase 3

All questions were translated from Dutch to English for the purpose of publication of this article.

**General user-friendliness**

1.      ‘How did you like using the platform?’

2.      ‘Have you encountered any problems while using the platform?’

**General helpfulness platform**

1.      [ranking exercise] ‘Which part of ENYOY was most helpful to you?’

a.      ‘Why was this the most helpful?’

b.      ‘Why were the other parts less helpful?’

c.      ‘Why would you put [...] first?’

**Community**

**Therapy exercises**

**Coaching calls**

**Value/ helpfulness assignments: Journeys, explore function and toolkit**

1.   ‘What do you think of the ‘therapy journey’?’ (By this I mean the journey that is/was set up for you)

a.     ‘Why do you think that?’

b.      ‘How much did you use the therapy journey? Why or why not?’

- ‘Did you run into things that made you use the journey less?’

c.      ‘To what extent has your therapy journey helped you? /How valuable is the therapy journey to you?’

2.    ‘Did you use the ‘explore function’?’ (The page where you see all the available exercises)

a.      ‘Why did you use the explore function?’

- ‘To what extent have these assignments helped you?’

b.      ‘How come you did not use it?’

- ‘What should change in order for you to use it?’

3.      ‘What do you think of the ‘toolkit’?’

a.     ‘Why do you think that?’

b.     ‘How important is it for you that you can save assignments in the toolkit?’

c.      ‘Have you ever saved things in the toolkit?’

- ‘Why?’

d.      ‘Have you ever used exercises from your toolkit?’

- ‘Why is that?’
- ‘How did you feel after performing such an exercise?’

e.      ‘How come you did not use it?’

- ‘What should change in order for you to use it?’

**Value/ helpfulness community**

1.      ‘What do you think of the ‘community’?’

1. ‘How do you feel about being able to read other people's posts?’

- In what ways did reading messages from others help you?

1. ‘Have you ever posted yourself?’

- ‘What was that like for you?’
- ‘How did you feel after posting?’
- ‘To what extent has posting helped you?’

1. ‘Have you ever posted comments yourself?’
   - ‘What was that like for you?’
   - ‘How did you feel after posting?’
   - ‘To what extent has posting helped you?’
2. ‘Why is it that you have not posted anything in the community?’
   - ‘What should change in order for you to use it?’

[**Rating exercise**]

1. ‘On a scale of 1 to 10 how helpful do you find this part, so to what extent do you think this part helps you / has helped you at ENYOY?’
2. ‘Why do you give this part this rating?’
