# Supplementary material for: Moderated digital social therapy for young people with emerging mental health problems: A user-centered mixed-method design and usability study
Source: Front Digit Health. 2023 Jan 9;4:1020753. doi: 10.3389/fdgth.2022.1020753 (PMC9869113; doi:10.3389/fdgth.2022.1020753)
Supplement: Supplementary file 4 [file Datasheet4.docx]

**Appendix 4**. Completion rates and mean completion time per task

| Task | | Subtask | Completion rate | | | Prompts | Mean completion time s (*SD*) | | Range completion time s | |
| --- | --- | --- | --- | --- | --- | --- | --- | --- | --- | --- |
|  |  |  | CTA | RTA | Overall | Overall | CTA | RTA | CTA | RTA |
| 1 | Log on to ENYOY (a)  and hide profile and messages (b) | a | 5/5 | 5/5 | 10/10 | 7 | 234 (93) | 91 (64) | 128-347 | 20-168 |
|  |  | b | 5/5 | 5/5 |  | 5 | 110 (143) | 58 (42) | 18-363 | 23-123 |
|  |  | Overall | 5/5 | 5/5 |  | 12 | 344 (236) | 148 (106) | 18-363 | 20-168 |
| 2 | Request a different therapy journey |  | 5/5 | 4/5 | 9/10 | - | 106 (51) | 147 (56) | 49-177 | 9-339 |
| 3 | Find comics for ‘Piekeren’ in the explore function |  | 5/5 | 4/5 | 9/10 | - | 86 (15) | 58 (30) | 62-100 | 25-93 |
| 4 | Save an exercise (a)  and find saved exercise (b) | a | 5/5 | 4/5 | 9/10 | - | 48 (43) | 39 (23) | 14-121 | 14-60 |
|  |  | b | 5/5 | 3/5 | 8/10 | - | 42 (28) | 15 (5) | 13-82 | 10-19 |
|  |  | Overall | 5/5 | 3/5 | 8/10 | - | 90 (71) | 54 (28) | 13-121 | 10-60 |
| 5 | Send a chat request to a moderator or peer worker |  | 4/5 | 4/5 | 9/10 | 2 | 74 (84) | 99 (68) | 12-198 | 18-183 |
| 6 | Find tips for when you feel distressed |  | 5/5 | 4/5 | 9/10 | 1 | 25 (30) | 45 (66) | 9-80 | 2-136 |
| 7 | Send a venting message in the community |  | 5/5 | 5/5 | 10/10 | - | 21 (12) | 29 (31) | 10-42 | 6-83 |
| 8 | Find a mindfulness exercise via the explore function |  | 5/5 | 4/5 | 9/10 | 1 | 71 (43) | 46 (41) | 33-132 | 18-105 |

*Notes*. CTA = concurrent think aloud method, RTA = retrospective think aloud method, s = seconds, *SD* = standard deviation
