# Supplementary material for: Moderated digital social therapy for young people with emerging mental health problems: A user-centered mixed-method design and usability study
Source: Front Digit Health. 2023 Jan 9;4:1020753. doi: 10.3389/fdgth.2022.1020753 (PMC9869113; doi:10.3389/fdgth.2022.1020753)
Supplement: Supplementary file 5 [file Datasheet5.docx]

**Appendix 5.** Suggestions for improvement per study phase

**Table a.** Overlapping suggestions for ENYOY resulting from all phases.

| Suggestion | Phase 1 | Phase 2 | Phase 3 |
| --- | --- | --- | --- |
| Being able to look at previously completed exercises | X | X | X |
| Receiving notifications to encourage usage of ENYOY and incorporate ENYOY in daily routine | X |  | X |
| Create uniformity in language on ENYOY (translate all content from English to Dutch, including comics) | X | X | X |
| Making ENYOY more accessible by simplifying the two-step verification log-in method. | X | X |  |

**Table b.** Unique suggestions for ENYOY resulting from all phases*

| Segment | Phase | Suggestion | Quote |
| --- | --- | --- | --- |
| Journey | 1 | Being able to switch to other therapy journeys without the approval of a clinical moderator. (++) | “So first I was assigned to the ‘anxiety’ journey, even though we sort of agreed that I, ehm, was more fit for the ‘depression’ box […] and that annoyed me a bit then” |
|  |  | A ‘table of contents’ including short summaries of the information that will be discussed in each therapy journey and with the possibility to skip items  (++) |  |
|  |  | Adding a feature where participants can see a summary of their main mental health complaints (++) |  |
|  | 3 | Incorporating ENYOY more often in coaching calls, to reduce participants viewing the platform and its journeys as too rigid (+) |  |
| Community | 2 | Adding a prompt for the ‘I am venting’-functionality, to motivate participants talk about difficult feelings (e.g. “I want to get something off my chest..”) (++) |  |
|  |  | Improve visibility of button to turn on/off  “venting”-messages of other participants (++) |  |
|  | 3 | Keep participants engaged with Community to lower the threshold to post personal messages. (++) |  |
| General use | 1 | A digital ‘post-it’ to write down notes (++) |  |
|  | 2 | Making the ENYOY website easier to find (+) | “I’ve always had to open it [red: ENYOY-platform] via my email, and logging in took me about half an hour.” |
|  | 3 | Being able to use an ENYOY application for easy access on a mobile device (++) | “I just forgot that it [red: 'ENYOY'] even existed. It was not in my system at all. It is a shame that there is a platform but not an app or something like that." |
| Explore | 2 | Add a search toolbar to increase ease of finding information (++) | “I actually think it’s a shame there isn’t a search function in the platform itself.. sometimes I didn’t know a concept very well, which made it even more difficult to find what I was looking for.” |
|  |  | Check whether exercises are coupled to right themes (e.g. exercise “rumination” is not coupled to the theme “rumination”) (++) |  |
|  |  | Adding a mindfulness-category for easy access (++) |  |
| Chat messages | 1 | Being able to contact others directly via chat (++) |  |
|  | 2 | Move message-function to Community (++) | “The first place I would look to message a peer worker would be the Community.” |
| “Stress”  -button** | 2 | Reduce text to create an overview. (++) | “This wall of text…. I’d get distracted, but yeah, that’s me” |
|  |  | Adding questions regarding the specific situation a participant is in (e.g. “Do you think of suicide? > call 113” / “Is  this a medical emergency? > call 112”) (+) |  |
|  |  | Add categories to create an overview. (++) | “I’m just saying that using categories would make it easier to see what’s relevant.” |

*notes.* * Suggestions from deducted data are marked with ‘+’, suggestions received directly from participants are marked with ‘++’ ** The Stress-button is a functionality on ENYOY when a young person is distressed and needs immediate medical attention (e.g. due to suicidal ideation, a medical emergency or an unsafe domestic situation).
