# Supplementary material for: Moderated digital social therapy for young people with emerging mental health problems: A user-centered mixed-method design and usability study
Source: Front Digit Health. 2023 Jan 9;4:1020753. doi: 10.3389/fdgth.2022.1020753 (PMC9869113; doi:10.3389/fdgth.2022.1020753)
Supplement: Supplementary file 6 [file Datasheet6.docx]

**Appendix 6.** Distribution of usability problems by problem type and severity per think-aloud method

|  | Usability problem | Location | Task | Detected by # evaluators (method) | Severity rate | Problem type |
| --- | --- | --- | --- | --- | --- | --- |
| 1 | User is unable to find the ENYOY-platform website without help (e-mail with a website link or link send in chat while interviewing) | Google search bar | 1 | 5 out of 5 (C)  2 out of 5 (R) | 3 | O |
| 2 | User confuses the system's login pop-up window data entry field with their personal login field (two-way authentication) | Log in – pop-up | 1 | 3 out of 5 (C)  1 out of 5 (R) | 3 | V |
| 3 | User needed to refresh the website for the pop-up window to appear | Log in – pop-up | 1 | 1 out of 5 (C) | 2 | N |
| 4 | User expected the 'edit profile' button under the 'profile' page. ‘Edit profile' button located under the profile image was not found | Profile, edit profile | 1a | 2 out of 5 (C) | 2 | N |
| 5 | User pressed 'adjust therapy journey’ instead of  'change therapy journey ' | Home - ‘change your therapy journey’ | 2 | 2 out of 5 (C)  1 out of 5 (R) | 2 | T |
| 6 | The user thinks the therapy journey is the same as the exercises found on the explore function page | Home, explore function | 2 | 3 out of 5 (C) | 2 | T |
| 7 | The platform is designed for Dutch native speakers however some texts are written in English, which could cause comprehension problems | Whole platform, mainly in exercises in explore function | 3 | 1 out of 5 (C) | 3 | O |
| 8 | User has difficulties finding a specific type of task in the explore function. (i.e., Mindfulness and 'Piekeren’ exercise) | Explore function | 3, 8 | 1 out of 5 (C)  2 out of 5 (R) | 2 | N |
| 9 | 'Do-it-later' button did not save the exercise, this resulted in a loss of liked/helpful exercise | Tasks – ‘Do-it-later’ | 4 | 1 out of 5 (C)  2 out of 5 (R) | 2 | E |
| 10 | User was unable to locate the saved ‘do-it-later’ exercise. | Home or Toolkit | 4a | 2 out of 5 (C)  1 out of 5 (R) | 2 | N |
| 11 | User did not understand the terms ‘Peer worker’ or ‘Enyoy team' and was wary in making contact for not knowing who is part of the team | Messages | 5 | 2 out of 5 (C) | 2 | T |
| 12 | User did not easily find the option to chat with a peer worker | Messages | 5 | 1 out of 5 (C) | 2 | N |
| 13 | User expects the function to chat anonymously with staff on the Community page (user will search ‘Community’ before ‘Messages’) | Community | 5 | 2 out of 5 (C)  4 out of 5 (R) | 3 | N |
| 14 | The meaning of the 'stress' button is unclear. The implication of severity (i.e., stressful situation where external help is needed) could result in users missing vital information in times of need | Stress | 6 | 1 out of 5 (C)  2 out of 5 (R) | 3 | T |
| 15 | User has trouble with the length of the text on the ‘Stress’ page. The page lacks a clear overview of the available options | Stress | 6 | 2 out of 5 (C) | 1 | V |
| 16 | The user expects 'change your therapy journey’ button in the 'settings' of your profile (it resulted that the user was unable to find the button) | Home –‘change therapy journey’ | 2 | 1 out of 5 (R) | 2 | O |
| 17 | It was unclear to the user that ‘change your therapy journey’ was clickable. | Home – change your therapy journey | 2 | 2 out of 5 (R) | 2 | V |
| 18 | It was unclear to the user what the logo and labels under explore function exercises meant. This resulted in not selecting the right task (e.g., ‘Reflective action’ versus  ‘Discussion point’) | Explore function – logo and labels under exercise | 3, 8 | 3 out of 5 (R) | 2 | G |

*Notes.* # = number, C = concurrent think aloud method, R = retrospective think aloud method, severity rate (Nielsen, 1994), V = visibility problems, O = overall ease of use, E = error messages/help instructions, T = terminology interpretation problems/meaning of labels, G = unclear graphics/symbols, N = navigation
